# Amygdala Perfusion Is Predicted by Its Functional Connectivity with the Ventromedial Prefrontal Cortex and Negative Affect

**DOI:** 10.1371/journal.pone.0097466

**Published:** 2014-05-09

**Authors:** Garth Coombs III, Marco L. Loggia, Douglas N. Greve, Daphne J. Holt

**Affiliations:** 1 The Department of Psychiatry, Massachusetts General Hospital, Boston, Massachusetts, United States of America; 2 The MGH/HST/MIT Athinoula A. Martinos Center for Biomedical Imaging, Charlestown, Massachusetts, United States of America; 3 Center for Brain Science, Department of Psychology, Harvard University, Cambridge, Massachusetts, United States of America; 4 Harvard Medical School, Boston, Massachusetts, United States of America; 5 The Department of Radiology, Massachusetts General Hospital, Boston, Massachusetts, United States of America; Bellvitge Biomedical Research Institute-IDIBELL, Spain

## Abstract

**Background:**

Previous studies have shown that the activity of the amygdala is elevated in people experiencing clinical and subclinical levels of anxiety and depression (negative affect). It has been proposed that a reduction in inhibitory input to the amygdala from the prefrontal cortex and resultant over-activity of the amygdala underlies this association. Prior studies have found relationships between negative affect and 1) amygdala over-activity and 2) reduced amygdala-prefrontal connectivity. However, it is not known whether elevated amygdala activity is associated with decreased amygdala-prefrontal connectivity during negative affect states.

**Methods:**

Here we used resting-state arterial spin labeling (ASL) and blood oxygenation level dependent (BOLD) functional magnetic resonance imaging (fMRI) in combination to test this model, measuring the activity (regional cerebral blood flow, rCBF) and functional connectivity (correlated fluctuations in the BOLD signal) of one subregion of the amygdala with strong connections with the prefrontal cortex, the basolateral nucleus (BLA), and subsyndromal anxiety levels in 38 healthy subjects.

**Results:**

BLA rCBF was strongly correlated with anxiety levels. Moreover, both BLA rCBF and anxiety were inversely correlated with the strength of the functional coupling of the BLA with the caudal ventromedial prefrontal cortex. Lastly, BLA perfusion was found to be a mediator of the relationship between BLA-prefrontal connectivity and anxiety.

**Conclusions:**

These results show that both perfusion of the BLA and a measure of its functional coupling with the prefrontal cortex directly index anxiety levels in healthy subjects, and that low BLA-prefrontal connectivity may lead to increased BLA activity and resulting anxiety. Thus, these data provide key evidence for an often-cited circuitry model of negative affect, using a novel, multi-modal imaging approach.

## Introduction

One commonly cited model of mental illness is derived from the writings of the neurologist Hughlings Jackson, who observed that frontal lobe lesions were associated with ‘release behaviors’, i.e., disinhibition of suppressed, primitive reflexes that are generated subcortically [1], [2]. Based on Jackson’s ideas, theories about circuitry abnormalities underlying a diverse set of disorders, including depression [3], [4], Post-Traumatic Stress Disorder [5], and schizophrenia [6], [7], have suggested that disruptions of projections from the prefrontal cortex to subcortical structures lead to a loss of inhibitory control and over-activity of these structures, with the subsequent emergence of symptoms. Neuroimaging research over the past several decades has generated support for this model [8]–[11], most consistently with demonstrations of associations between increases in negative affect (i.e., anxiety, depression, or subsyndromal expressions of those symptoms [12]) and 1) increased responses [13] or resting activity [14], [15] of the amygdala, or 2) decreased strength of the connections between the prefrontal cortex and amygdala [16]–[18]. However, it has remained unclear whether decreased prefrontal-amygdala connectivity is associated with amygdala over-activity during these states.

A novel approach to testing this model is to combine two MRI-based methods that measure “resting-state” brain activity: 1) arterial spin labeling (ASL) functional MRI (fMRI), which uses magnetically-labeled arterial blood water as an endogenous tracer, providing a quantitative measure of regional cerebral blood flow (rCBF), and 2) blood oxygenation level-dependent (BOLD) functional connectivity fMRI, which measures correlations in spontaneous, low frequency fluctuations in the BOLD signal between different brain regions [19]. ASL fMRI has been validated by comparisons with positron emission tomography measurements of rCBF at rest [20]. It is particularly well-suited for the quantitative measurement of tonic neural processes, since it is relatively free of the slow drift associated with BOLD contrast imaging, and has good reliability [20], [21]. Resting-state BOLD functional connectivity, on the other hand, is now widely used as a measure of the degree of functional coupling among distinct brain regions comprising a network [22].

Previous studies using resting-state BOLD data to measure functional connectivity have shown that the basolateral nucleus of the amygdala (BLA) in humans displays a high degree of functional correlation at rest with the ventral medial prefrontal cortex (mPFC), whereas the central nucleus of the human amygdala shows little connectivity with the mPFC [23], [24], similar to the pattern of amygdala projections found in non-human primates [25], [26]. In the present study, we first tested whether ASL fMRI could detect a relationship between one type of negative affect, current anxiety levels, and BLA activity. We then used ASL and BOLD fMRI in combination to test the hypothesis that elevated BLA rCBF is associated with increased anxiety and reduced BLA-prefrontal connectivity.

## Materials and Methods

### Ethics Statement

Written informed consent was obtained from all subjects prior to enrollment, in accordance with the guidelines of the Partners HealthCare Institutional Review Board, which approved this study.

### Subjects and Anxiety Measure

38 healthy adults (11 females; see Table 1 for demographic information) were recruited via advertisement. Subjects were excluded if they had a current psychiatric disorder or history of one, including a history of substance abuse or dependence, or who had used illicit substances within the three months prior to the study, as determined by the Structured Clinical Interview for DSM-IV (SCID) [27]. Subjects were also excluded if they had ever used psychotropic medications, had any contraindications for MRI scanning (metal implants, claustrophobia), or had a neurologic disorder or a history of head trauma with neurologic sequelae. Four subjects did not have resting BOLD scans and thus were not included in the functional connectivity MRI analyses. Four subjects did not have ASL scans and thus were not included in the ASL analyses. One additional subject was excluded from the ASL analyses due to poor data quality (movement >3 mm). The Spielberger State and Trait Anxiety Inventory (STAI) [28] was administered on the same day that the scanning occurred. Factor analyses have shown that the STAI measures symptoms typically associated with depression (e.g., anhedonia), as well as those associated with anxiety; thus it appears to index the broad construct of negative affect, rather than anxiety only [29]–[34]. Consistent with this, it has been found that scores on the STAI are equally or more highly correlated with measures of depression than with scores on other anxiety scales [32]. However, since we used just one measure of negative affect (the STAI state measure), we have referred to the data collected with the STAI here as “anxiety levels”.

**Table 1:** Participant demographics.

	Mean (SD)	Range (Min, Max)
Age (years)	29.7 (9.9)	19, 53
Education Level (years completed)	15.7 (1.5)	12, 19
Verbal IQ^a^	111.2 (6.1)	97.6, 125.1
Anxiety (state)^b^	25.2 (4.4)	20, 37
Anxiety (trait)^b^	27.4 (7.1)	20, 47
Socioeconomic Status^c^	47.3 (8.6)	32, 66
Depression^d^	1.1 (1.7)	0, 6
Caucasian/Non-Hispanic (%)	78.9	
Caucasian/Hispanic (%)	7.9	
Black/African-American (%)	7.9	
Asian (%)	5.3	

Participant demographics are listed. Values given are Mean (Standard Deviation) unless otherwise specified.

a Verbal IQ was measured using the North American Adult Reading Test [74].

b Anxiety was measured using the Spielberger State and Trait Anxiety Inventory [28].

c Socioeconomic Status was measured using the Hollingshead Scale [75].

d Depression was measured using the Beck Depression Inventory [76].

### Image Acquisition

Imaging took place in a 3-Tesla Siemens TIM Trio MRI scanner (Siemens Medical Systems, Erlangen, Germany) using a 12-channel head coil. Subjects underwent two conventional high-resolution three-dimensional structural T1 magnetization prepared rapid acquisition gradient-echo (MPRAGE) scans (8 min 7 sec, 128 sagittal slices, 1.33-mm thickness, repetition time (TR) = 2530 ms, echo time (TE) = 3.39 ms, flip angle = 7°, resolution 1.3×1×1.3 mm).

One 6-min-2-sec pulsed ASL scan was collected using the “PICORE-Q2TIPS” MRI labeling method [35], with the following parameters: TR = 3000 ms, TE = 13 ms, TI1 = 700 ms, TI2 = 1700 ms, voxel size = 3.515×3.515×6.25 mm, number of slices = 16 (5 mm thickness with a 1.25 mm gap), FOV = 22.5 cm, matrix = 64×64, flip angle = 90 degrees. ‘Tag’ images were acquired by labeling a thick inversion slab (110 mm), proximal to the imaging slices (gap = 21.2 mm). 57 ‘Control’ images were acquired interleaved with 57 tag images by applying an off-resonance inversion pulse without any spatial encoding gradient. At the beginning of each ASL scan, an M_0_ scan (i.e., the longitudinal magnetization of fully relaxed tissue) was acquired for resting regional cerebral blood flow (rCBF) quantification purposes [36]. A delay time of 1 second was inserted between the end of labeling pulses and image acquisition to reduce transit-related effects.

One 6-min-12-sec resting BOLD scan (TR = 3000 ms; TE = 30 ms; flip angle = 85 degrees; 47×3 mm thick slices, 3×3×3 mm in-plane resolution) was also acquired.

During both the ASL and BOLD scans, subjects were instructed to keep their eyes open, blink normally, and look at a blank screen.

### Regions-Of-Interest

Regional CBF levels were extracted from the basolateral nucleus of the amygdala (BLA) using an anatomically-defined BLA region-of-interest (ROI). The BLA ROIs were derived from the Jeulich Histological Atlas [37], as in previous studies of amygdala functional connectivity [23], [24]. The ROIs were comprised of voxels with ≥50% probability of being located in the BLA. Voxels near a subregion border were included if they were more likely to be located in the BLA than in other amygdala subregions [23]. For the ASL analyses, these ROIs were then automatically mapped into each individual subject’s native anatomical scan. To maximize power, a seed combining the left and right BLA ROIs was created and used as the seed for the functional connectivity fMRI analyses.

### ASL fMRI Analysis

ASL data analyses were performed using a combination of FSL (FMRIB’s Software Library, www.fmrib.ox.ac.uk/fsl) [38] and FreeSurfer (http://surfer.nmr.mgh.harvard.edu/) tools. The ‘tag’, ‘control’, and M_0_ scans were first motion-corrected using MCFLIRT [39] and registered to the high resolution anatomical images using FreeSurfer’s bbregsiter tool [40]. Then tag and control scans were surround subtracted (i.e. given each tag_X_, [(control_X−1_+control_X+1_)/2−tag_X_]) to generate perfusion-weighted images. All of the perfusion-weighted maps were then averaged and scaled by a factor proportional to the M_0_ scan to obtain rCBF maps in absolute values (ml/100 g of tissue/min) [36]. Group-level analyses were conducted in the volume, and group rCBF maps were smoothed with a 3D isotropic, 7.02 mm full-width half-maximum (FWHM) Gaussian kernel (set to 2*voxel size). Regional CBF values were extracted from the BLA ROIs mapped into each subject’s individual anatomical scan. Additionally, whole-brain voxel-wise analyses using STAI state anxiety score as a regressor were conducted, in order to determine whether there were significant correlations between anxiety and rCBF of regions other than the amygdala. In this analysis, the Gaussian Random Fields (GRF) method was used to correct for multiple comparisons [41], with a voxel-level height threshold of p<.0005. Clusters that showed cluster-wise significance (cluster-level threshold of p<.05, GRF whole brain corrected) are reported in the text and in Table 2.

**Table 2:** Regions showing a correlation between perfusion and anxiety levels.

Region	BA	Hemi	Tal (x, y, z)	Size (mm^3^)	Z
Superior frontal gyri	9	R	9, 50, 25	7811	5.0
Superior parietal gyrus, precuneus	7	R	23, −72, 34	1197	4.5
	7	R	14, −52, 50	1771	4.4
Thalamus, hypothalamus, posterior hippocampus		L	−11, −20, −1	6714	4.5
		R	13, −21, −1	3002	4.2
Putamen (posterior), hippocampus, amygdala		L	−34, −17, −7	3283	4.5
Putamen (anterior)		L	−11, 19, −11	1769	4.5
Postcentral gyrus	41	L	−48, −19, 23	1145	4.4
	43	R	60, −11, 13	948	4.0
Middle frontal gyrus	46	R	30, 45, 24	2286	4.3
Posterior cingulate gyrus	23	L	−5, −43, 24	3504	4.3
Rostral anterior cingulate, medial frontal gyri	32/9	L	−7, 47, 11	525	4.1
Hippocampus, putamen		R	41, −19, −9	2094	4.0

Results of a whole-brain voxel-wise regression analysis of the regional cerebral blood flow (rCBF) data, using anxiety levels as a regressor, are listed. Sites which showed a significant positive correlation between rCBF and anxiety levels are listed below. Also, see Figure 2. BA = Brodmann Area; Hemi = hemisphere; Tal = Talaraich coordinates.

### Functional Connectivity fMRI Analysis

Standardized preprocessing techniques [42] were used to selectively capture variance in the BOLD signal corresponding to low-frequency (<.08 Hz) fluctuations in neural activity. Data were smoothed using a 7 mm FWHM Gaussian kernel. Nuisance regressors, including the six parameters computed from the rigid-body motion correction, the averaged signal within a ventricular ROI, a region within the deep white matter, and the signal averaged over the whole brain, were used to remove systematic variance associated with these variables. The first temporal derivative of each regressor was also included to account for temporal shifts in the BOLD signal. Measures of slice-based and voxel-based signal-to-noise ratios (SNR) and the following motion parameters [43] were calculated for each BOLD scan to test if these parameters correlated with anxiety or perfusion levels: 1) Mean relative translations in 3D space (each volume compared to the previous timepoint), 2) Mean absolute translations in 3D space (each volume compared to the first timepoint), 3) Number of micro-movements (the number of relative translations in 3D space >.5 mm), and 4) Mean absolute rotations in the x, y, and z directions.

To create whole-brain correlation images, the averaged time series across all voxels comprising the seed (the bilateral BLA ROI) was used as the variable of interest in a linear regression with the time series corresponding to each voxel across the brain. All statistical analyses of correlation data were performed on Fisher z transforms [44]. Only clusters that reached whole-brain family-wise error (FWE) significance with a height threshold of p<.05 and size threshold of ≥5 voxels are reported (Table 2).

Whole-brain random effects multiple regression analyses using STAI state anxiety score or rCBF of the BLA as effects of interest were conducted to identify locations showing correlations between the strength of their connectivity with the seed and the anxiety or BLA rCBF measures. Analyses were restricted to regions of the brain that showed significant connectivity with the seed region (either positive or negative). Based on previous work showing that the BLA is highly anatomically and functionally connected with the ventral mPFC (see Introduction), and a recent meta-analytic factor analysis suggesting that the ventrocaudal mPFC is primarily involved in ‘affect generation’ [45], we chose the ventral mPFC as the *a priori* search area for the regression analyses. The search area was confined to a ventral mPFC mask (39,192 mm^3^) consisting of voxels showing significant BLA-mPFC functional coupling within arbitrary boundaries containing the ventral mPFC region: x-coordinate ≤|10|, y-coordinate ≥0, and z-coordinate ≤10. Thus this independent, *a priori* mask was used in order to restrict the subsequent analyses to voxels within the ventral mPFC showing functional connectivity with the BLA, prior to conducting the correlational analyses of interest. Sites showing correlations between BLA-mPFC connectivity and the regression variable (anxiety or rCBF) were deemed significant if they met a cluster-wise correction (FWE, p<.05) within the ventral mPFC. All second-level analyses were conducted using SPM5 (http://www.fil.ion.ucl.ac.uk/spm).

The location of significant clusters was determined using the Talairach and Tournoux Stereotaxic Atlas [46] and confirmed with the Wake Forrest University (WFU) PickAtlas [47].

### Secondary Mediation Analyses

A mediation analysis was then conducted to assess the relationships among the results of our regression analyses (i.e., the direction of those effects, with two possible outcomes or models of the relationships, see below). The relationship between BLA-mPFC connectivity strength, BLA perfusion levels, and anxiety scores was assessed using the Indirect Mediation Analysis script created by Preacher and Hayes for SPSS [48]. Non-parametric bias-corrected bootstrapping (with 10,000 bootstrap samples) was used to derive 95% Confidence Intervals of the indirect effects.

## Results

### Anxiety

The mean (± standard deviation) state anxiety score of this sample (25.2±4.4; median = 24, range 20–37, with a possible range of 20–80) was well below means typically reported in patients with anxiety and other psychiatric disorders [28], [49]; scores on the STAI-state of greater than 40 are considered clinically significant [49]. Thus, all subjects had STAI-state scores in the healthy to subsyndromal range. See Table 1 for additional details about the subjects.

### ASL Analysis

**Figure 1:**
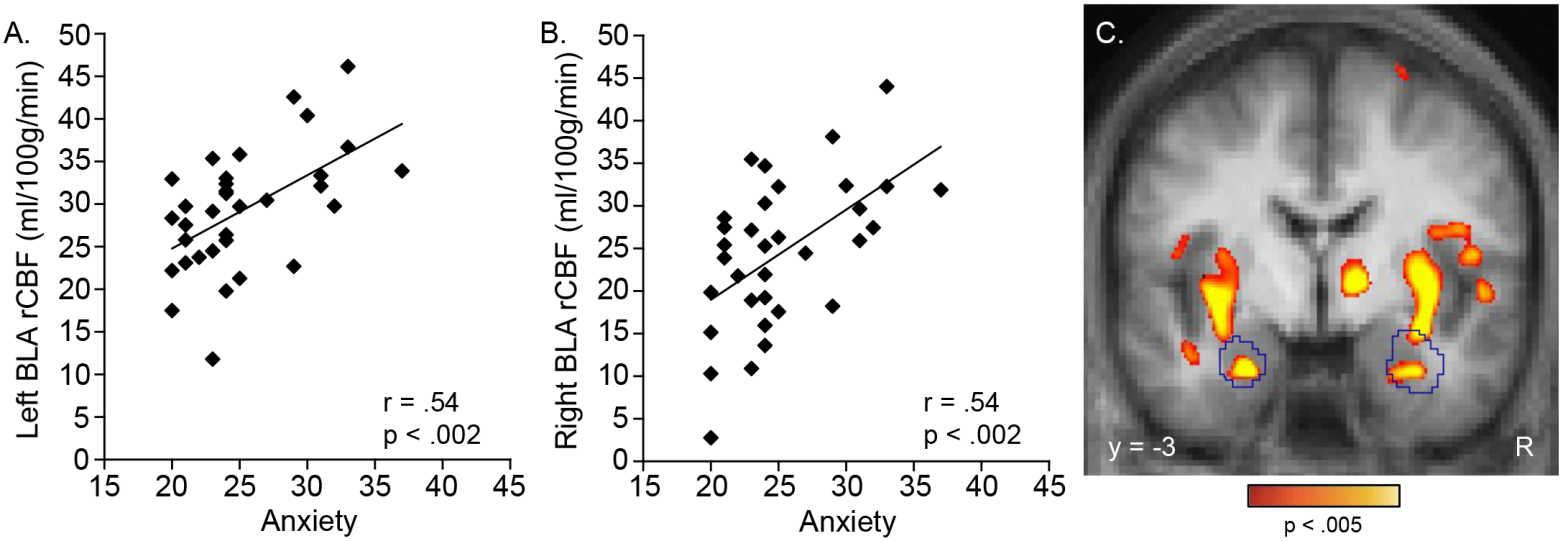
Perfusion of the basolateral nucleus of the amygdala (BLA) is correlated with anxiety levels. Significant correlations were found between anxiety levels and perfusion of the left (A) and right (B) basolateral amygdala (BLA), as defined using anatomical regions-of-interest. These findings were then confirmed in a voxel-wise, whole brain regression analysis (C). In C, the BLA regions-of-interest are outlined in blue; the voxel-level display threshold is p<.005 (showing only clusters surviving whole-brain correction, see Methods). Clusters that showed cluster-wise significance (p<.05, whole brain corrected) are reported in the text and in Table 2. R, right.

**Figure 2:**
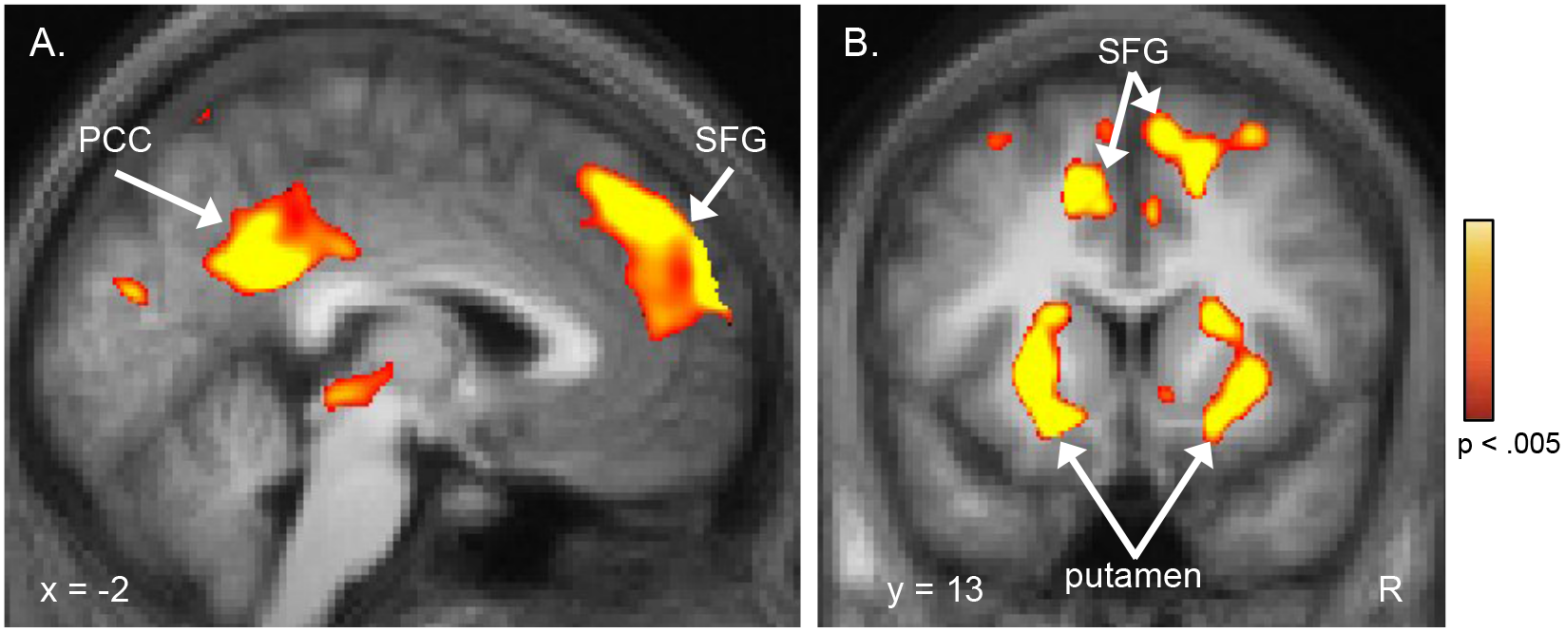
Perfusion of a distributed network of regions outside of the amygdala is also correlated with anxiety levels. A voxel-wise whole brain regression analysis revealed that, in addition to the basolateral amygdala (BLA), perfusion of the superior frontal gyri and posterior cingulate cortex (A), and anterior putamen (B), among other regions (see Table 2), were significantly correlated with anxiety levels. Whole-brain corrected results (see Methods) are displayed here using a voxel-level threshold of p<.005. Clusters that showed cluster-wise significance (p<.05, whole brain corrected) are reported in the text and in Table 2. R, right; PCC, posterior cingulate cortex; SFG, superior frontal gyri.

Perfusion of both the right and left BLA was significantly correlated with anxiety levels (both right and left BLA: r = .54, p<.002) (Figure 1A and 1B). This finding was then confirmed in a follow-up, voxel-wise, whole brain-corrected regression analysis, which revealed strong correlations between anxiety and perfusion of voxels within the right (x, y and z Talairach coordinates of peak voxel: 33, −5, −13; p<5×10^−4^) and left (−22, −10, −22; p<1×10^−4^) amygdala, the majority of which fell within the boundaries of the BLA ROIs (Figure 1C). In addition to the amygdala, the whole brain regression revealed that anxiety was also significantly correlated with rCBF of the superior frontal gyri, posterior cingulate cortex (PCC), thalamus, and putamen (Figure 2A and 2B), as well as the superior parietal gyrus and precuneus, postcentral gyrus, middle frontal gyrus, rostral anterior cingulate and medial frontal gyri, and hippocampus (Table 2).

### Resting-state Functional Connectivity Analysis

**Figure 3:**
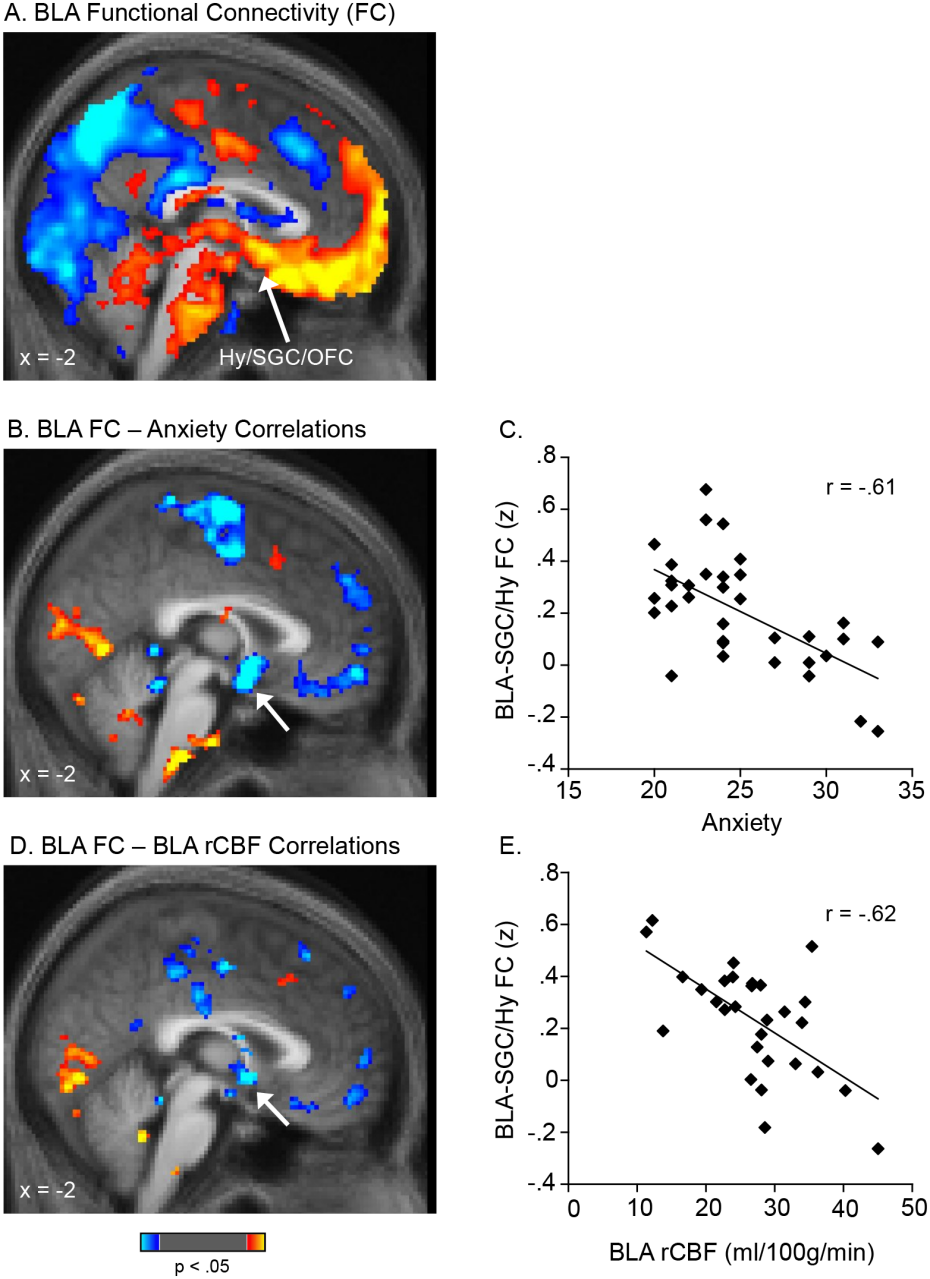
Functional connectivity between the BLA and mPFC is inversely correlated with BLA perfusion and anxiety levels. An average map of basolateral amygdala (BLA) functional connectivity is shown in A. A whole-brain voxel-wise regression revealed that the strength of connectivity between the BLA and mPFC was negatively correlated with both: anxiety levels (B, C) and BLA perfusion (D, E). In A, B, and D, voxels with positive connectivity with the BLA (A) or showing positive correlations between their connectivity with the BLA and anxiety levels (B) or BLA perfusion (D) are shown in warm colors; voxels with negative correlations are shown in cool colors. The scatter plots in C and E are derived from the accompanying voxel-wise regression maps shown in B and D and are presented for the purpose of illustrating the range of values only. Data are displayed at a threshold of p<.05. The clusters indicated with arrows in B and D met a cluster-wise correction (FWE, p<.05) within the ventral mPFC. The peaks of the clusters in B (4, 2, −7) and D (2, 4, −4) were localized to the posterior-most portion of the SGC (with both clusters extending into the hypothalamus) using two independent atlases (the Talairach and Tournoux Stereotaxic Atlas [46] and the Wake Forrest University (WFU) PickAtlas [47]; see Methods). Prior work further supports this localization; previously reported sites that have been localized to the SGC (BA25), as well as an architectonic mapping of BA25 [68], overlap with the two clusters reported here, with nearby peaks: 4, 2, −4 [69]; −2, 6, −6 [8]; −2, 8, −10 [70]; −3, 9, −6 [71]; −4, 9, −12 & 2, 11, −7 [72]; 0, 8, −16 [73]. BLA, basolateral amygdala; FC, functional connectivity; Hy, hypothalamus; SGC, subgenual cingulate gyrus; mPFC, medial prefrontal cortex.

An average map of the functional connectivity of the BLA confirmed [23], [24] that BLA activity is significantly coupled with the activity of the ventral mPFC, hypothalamus and brainstem (Figure 3A; Table 3).

**Table 3:** Basolateral amygdala functional connectivity.

Region	BA	Hemi	Tal (x, y, z)	Size (mm^3^)	Z
Medial orbitofrontal gyrus	11	L	−6, 19, −18	2160	6.4
	11	L	−6, 58, −11	40	5.4
Anterior medial orbitofrontal gyrus, frontal pole	10		0, 66, 3	152	5.7
Hippocampus		R	20, −33, 0	72	5.6
Insula		R	32, −7, 10	40	5.5
Lateral orbitofrontal gyrus	11	R	28, 28, −17	56	5.3
Inferior parietal gyrus	7	R	50, −45, 35	1816	6.5
Precuneus, posterior cingulate gyrus	7	R	4, −69, 51	4488	6.3
	7/31	R	4, −38, 46	264	5.5
	23	R	4, −28, 25	192	5.5
Inferior middle frontal gyri	10	R	40, 51, 3	3264	6.3
Superior, middle frontal gyri	8	L	−30, 31, 37	400	6.2
	9	R	38, 37, 33	1176	6.0
Lateral orbitofrontal gyrus	10/11	L	−24, 44, −7	168	6.0
	10/11	L	−24, 43, 2	40	5.2
Middle frontal gyrus	46	R	38, 44, 20	400	5.9
Dorsal anterior cingulate gyrus	32/8	R	4, 31, 35	296	5.5
Superior frontal gyrus	6	R	28, 10, 49	80	5.4
	6	R	24, 16, 54	88	5.4
Superior parietal gyrus	7	R	20, −67, 55	56	5.3

Areas of the brain showing significant functional connectivity with the basolateral amygdala (BLA) are listed. Clusters that are unshaded are those with positive functional coupling with the BLA, whereas clusters that are shaded grey are those showing negative functional coupling (inverse or anti-correlations) with the BLA (following global mean regression). Sites of connectivity within or abutting the BLA are not listed because of the difficulty of interpreting these findings. Also see Figure 3. BA = Brodmann Area; Hemi = hemisphere; Tal = Talaraich coordinates.

### Whole Brain Regression Analyses

Whole brain regression analyses revealed that anxiety was significantly inversely correlated with connectivity between the BLA and several regions of the mPFC, including the subgenual cingulate gyrus (SGC) (extending to the adjacent hypothalamus; 4, 2, −7; p_corrected_ <.02; 3392 mm^3^; Z = 4.4), orbitofrontal cortex, and superior frontal gyrus (Figure 3B). Moreover, BLA rCBF was also inversely correlated with connectivity between the BLA and the mPFC, including the SGC (extending to the adjacent hypothalamus; 2, 4, −4; p_corrected_ <.04; 3440 mm^3^; Z = 4.2) and medial frontal cortex (Figure 3D). These peaks were located in the posterior-most portion of the SGC bordering on the hypothalamus [46], [47]. In summary, lower BLA-mPFC functional connectivity was associated with both greater BLA rCBF and greater anxiety.

None of the motion parameters (relative or absolute head displacement, head rotations, or micro-movements) or SNR measures correlated with anxiety or perfusion (all rs<.32, all ps>.10).

### Mediation Analysis

**Figure 4:**
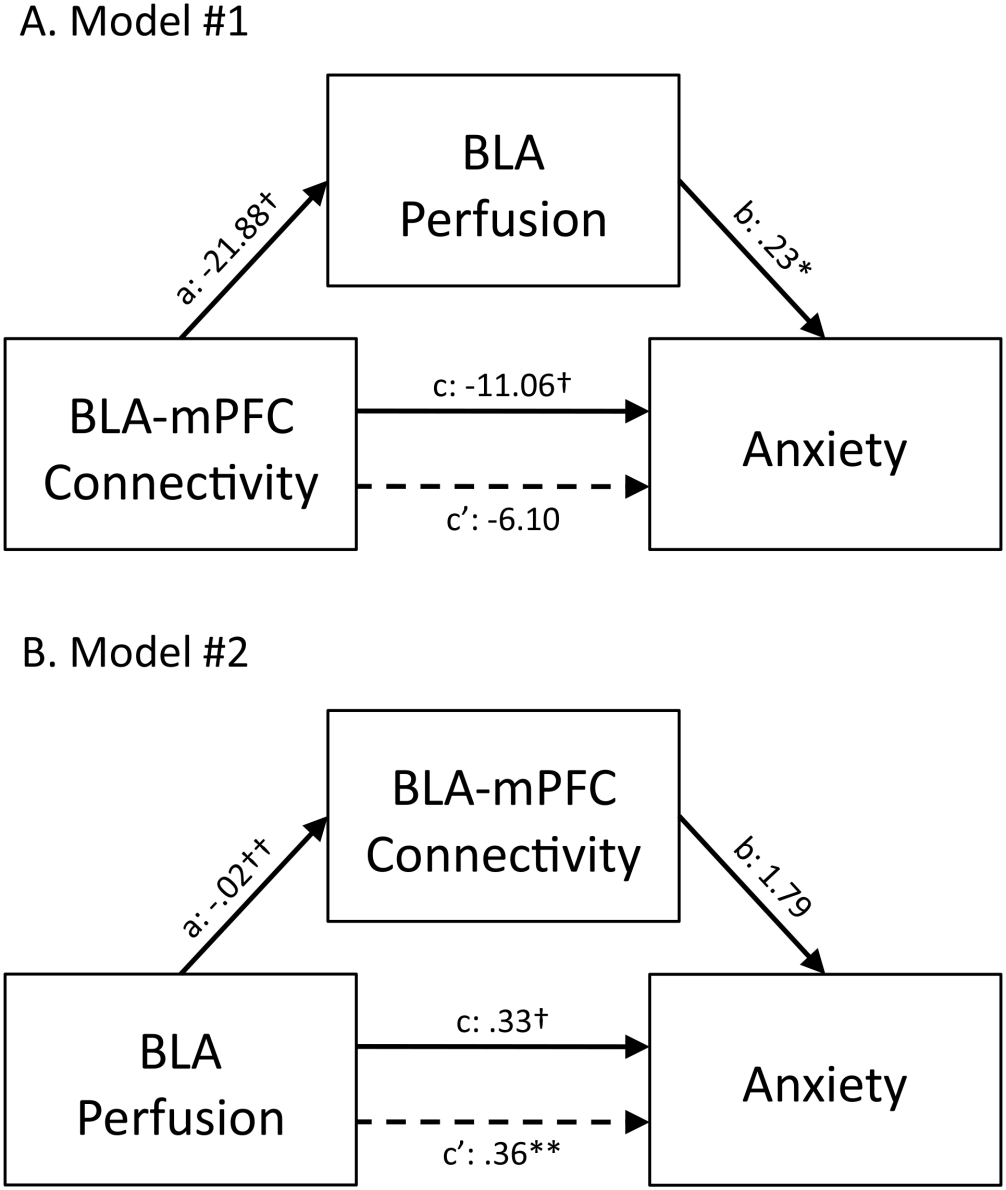
BLA perfusion mediates the relationship between BLA-mPFC connectivity and anxiety levels. Mediation analyses revealed that BLA perfusion levels mediate the relationship between BLA-mPFC connectivity and anxiety (A: Model #1). In contrast, BLA-mPFC connectivity did not mediate the relationship between BLA perfusion levels and anxiety (B: Model #2). Values are unstandardized regression coefficients reflecting the direct (paths a, b, and c’) and total (path c) effects of each relationship in the mediation model. BLA, basolateral amygdala; mPFC, medial prefrontal cortex; *p<.05, **p<.01, †p<.001, ††p<.0001.

A follow-up, secondary mediation analysis was then conducted to assess the relationship among the findings of the regression analyses conducted above, to distinguish between the following two models (also see Figure 4): 1) BLA perfusion mediates the relationship between BLA-mPFC connectivity and anxiety (Model #1), or 2) BLA-mPFC connectivity mediates the relationship between BLA perfusion and anxiety (Model #2).

To conduct this analysis, BLA-mPFC connectivity levels that had been found to correlate with anxiety or BLA perfusion were extracted for each subject in the following manner: The time course of mPFC resting-state BOLD activity was extracted from 3 mm-radii spheres centered on the locations of the peak correlations with anxiety levels (for the test of Model #1) or BLA perfusion (for the test of Model #2), and then correlated with the time-course extracted from the anatomical BLA seed. These correlations were then transformed into z-scores for each subject, reflecting BLA-mPFC connectivity strength.

Testing of Model #1 (Figure 4A) revealed a significant direct effect of BLA-mPFC connectivity on perfusion levels (path a: −21.88±5.48 [unstandardized coefficient ± standard error], t = −3.99, p<.001), a significant direct effect of perfusion levels on anxiety (path b: .23±.09, t = 2.46, p<.05), and a significant total effect of BLA-mPFC connectivity on anxiety (path c: −11.06±2.86, t = −3.87, p<.001). However, when perfusion was taken into account as a mediating variable, the direct effect of BLA-mPFC connectivity on anxiety was no longer significant (path c’: −6.10±3.31, t = −1.84, p = .08), indicating a specific mediated effect. Additionally, the bias-corrected bootstrapping test of the indirect effects of BLA-mPFC connectivity on anxiety through BLA perfusion as a mediator (path ab: −5.44±3.05) reached significance, with the 95% confidence intervals (−13.38, −1.27) indicating a specific mediated effect. In other words, BLA perfusion levels accounted for a significant portion of the relationship between BLA-mPFC connectivity and anxiety.

Testing of Model #2 (Figure 4B) revealed a significant direct effect of perfusion levels on connectivity (path a: −.02±.003, t = −6.04, p<1×10^−4^), a significant total effect of perfusion levels on anxiety (path c: .33±.08, t = 4.33, p<.001), but a non-significant direct effect of connectivity on anxiety (path b: 1.79±4.58, t = .37, p = .71). Additionally, the direct effect of perfusion levels on anxiety remained significant after taking into account BLA-mPFC connectivity strength (path c’: .36±.12, t = 3.06, p<.01), indicating that the relationship between BLA perfusion and anxiety is not mediated by BLA-mPFC connectivity. Furthermore, the bias-corrected bootstrapping test of the indirect effects of BLA perfusion on anxiety through BLA-mPFC connectivity as a mediator (path ab: −.03±.10) did not reach significance, with the 95% confidence intervals (−.21, .18) failing to indicate a specific mediated effect. In other words, BLA-mPFC connectivity failed to account for a statistically significant portion of the relationship between BLA perfusion levels and anxiety. In sum, these mediation results support Model #1, that reduced BLA-mPFC connectivity leads to elevated BLA perfusion and subsequent increases in anxiety, rather than the alternative possibility that elevated BLA perfusion leads to poor BLA-mPFC connectivity and increases in anxiety (Model #2).

## Discussion

The goal of this study was two-fold. First, we aimed to determine whether ASL fMRI could detect changes in amygdala activity related to variation in negative affect in healthy subjects. We found strong correlations between the perfusion of the BLA, as well as the hippocampus, putamen, and multiple midline frontal and parietal cortical areas, and anxiety levels, suggesting that ASL is a sensitive tool for measuring this relationship. Second, we sought to test a widely-cited model of psychopathology–the proposal that certain symptoms, including clinical anxiety and depression (which are thought to exist on a continuum with subsyndromal, everyday anxiety and other negatively-valenced affective states [50]–[52]), arise from dysregulation and resulting over-activity of subcortical structures such as the amygdala. This over-activity has been proposed to arise from a reduction in inhibitory input from the prefrontal cortex. Here, using advanced MRI-based techniques and data collected in healthy subjects who have not been exposed to potentially confounding treatments and chronic illness, we detected significant associations among all three components of this model, reduced prefrontal-subcortical (BLA) connectivity, subcortical (BLA) over-activity, and anxiety, in the same subjects.

Our finding of a strong correlation between anxiety levels and BLA perfusion are consistent with prior Positron Emission Tomography findings of associations between depression severity and the magnitude of amygdala blood flow [14] or metabolism [15] in patients with Major Depressive Disorder, and associations between levels of negative affect and amygdala blood flow in healthy subjects [53]. Also, task-based fMRI studies have found that the magnitude of amygdala BOLD responses is correlated with anxiety levels in healthy subjects [54]–[56]. The present findings reveal that ASL fMRI can also be used to detect and measure this relationship, providing a means of measuring the association that does not require exposure to radiotracers or a capacity to attend and respond to experimental stimuli.

Secondly, the results of the whole-brain regression analyses conducted here replicate and extend previous work showing that subsyndromal negative affect (measured with the same scale used here or with measures of overlapping constructs such as neuroticism) is predicted by the strength of amygdala-mPFC connectivity. Pezawas and colleagues first reported that amygdala-mPFC connectivity, as measured by task-elicited BOLD responses, is inversely correlated with temperamental negative affect [16]. This finding was later replicated using resting-state connectivity methods [17]. Also, Kim and Whalen used diffusion tensor imaging to show that anxiety levels are inversely correlated with the structural integrity of a white matter pathway overlapping with the expected trajectory of the uncinate fasciculus [18], a fiber tract containing projections between the amygdala and the subgenual cingulate and orbitofrontal gyri [57], [58]. This association may have other structural manifestations; for instance, negative affect levels have been correlated with increased amygdala volumes and decreased thickness of the left subgenual and rostral anterior cingulate cortices in healthy young adults [59]. Thus, our finding of an association between anxiety and amygdala-mPFC connectivity extends this body of previous work.

The present study shows for the first time that amygdala hyper-perfusion is linked to both anxiety and poor connectivity between the BLA and the caudal-ventral mPFC (BA25/subgenual cingulate cortex (SGC)) in the same subjects. Because conclusions cannot be drawn about the direction of these associations using regression alone, we used a mediation approach to determine whether, in our subjects, reduced connectivity strength within the BLA-mPFC pathway could be linked causally to elevated BLA perfusion and resulting anxiety. These analyses showed that the inverse relationship between BLA-mPFC connectivity and anxiety was significantly mediated by BLA perfusion levels, i.e., that decreased connectivity between the BLA and ventral mPFC contributes to BLA overactivity, resulting in higher anxiety levels. The alternative model, that BLA-mPFC connectivity mediates the relationship between BLA perfusion and anxiety levels, was not significant. Thus, these data provide further support for the Hughlings Jackson, top-down “release” model of negative affect–demonstrating associations among all three components of the model and providing preliminary evidence for a causal relationship between poor mPFC-amygdala connectivity and elevated amygdala activity and anxiety.

It should be noted that the mediation analysis in the current study was conducted using data extracted from the regression analyses; thus the findings only apply to this dataset. It will be necessary to repeat these analyses in an independent sample, and in clinically ill samples, to confirm these results. Also, the fact that the direct effect of BLA-mPFC connectivity on anxiety showed a trend towards significance after BLA perfusion was taken into account as a mediator suggests that additional factors, other than amygdala activity, influence the relationship between BLA-mPFC connectivity and anxiety. Follow-up studies can also consider the effects of other potential mediators on these relationships.

A model of the function of this pathway has been derived from findings of studies conducted in rodents. The infralimbic (IL) cortex in rodents sends glutamatergic inputs to the GABAergic intercalated cells of the amygdala, which, when stimulated, inhibit the output neurons of the central nucleus of the amygdala [60], [61]. Via this pathway, IL is able to inhibit, in a context-dependent manner, the fear-related autonomic responses generated by the central nucleus of the amygdala. There is some evidence from neuroimaging studies in humans that the human homologue of rodent IL is the SGC [62], [63], although some studies suggest that the homologue is located more rostrally in the mPFC [64], or that there is both a rostral and SGC portion of the mPFC that have a role in fear inhibition [62].

In the current study, resting BOLD activity of an extended region of the ventral mPFC was positively coupled with resting BOLD activity of the BLA, consistent with previous findings [23], [24] and closely mirroring the anatomical distribution of the amygdala-mPFC pathway seen in non-human primates [25], [26]. The portion of the BLA-mPFC circuit that was found to be weaker here (i.e., showing reduced BLA-mPFC coupling) in subjects with higher anxiety or BLA perfusion levels was the BLA-SGC pathway. In light of the animal literature and human imaging data indicating that fear inhibition is in part mediated by the SGC [62]–[64], our data suggest that the function of projections from the SGC to the inhibitory intercalated cells within the amygdala may be compromised during negative affect states, leading to elevated firing of the amygdala (and elevated perfusion) and increases in autonomic arousal and anxiety.

In the present data, the portion of the amygdala showing the greatest elevation in perfusion in relation to anxiety levels corresponded roughly (based on an atlas-based segmentation of the amygdala) to the BLA, rather than the central nucleus of the amygdala, suggesting that disruption of the SGC projection to the BLA (which is larger than the central nucleus in humans) may lead to its disinhibition, resulting in over-stimulation of the central nucleus and elevated arousal and anxiety. Since the GABAergic intercalated cells reside between the basolateral and central nuclei of the amygdala and influence the activity of both nuclei [65], this is one of several plausible mechanisms that could account for our results. Additional parallel work in humans and rodents will be needed to further test this possible interpretation of our findings.

In summary, these results show that ASL is a sensitive tool for objectively measuring the neural effects of subtle variation in anxiety levels. Given that ASL scans are non-invasive, brief and have excellent test-retest reliability, ASL could be used in the future as a clinical procedure which aids in the assessment of risk for future development of psychiatric illnesses, including severe anxiety disorders, such as PTSD, depression or other illnesses associated with impaired functioning of medial temporal lobe structures, such as schizophrenia. Secondly, we also found associations among individual variation in amygdala-mPFC functional coupling, amygdala perfusion, and anxiety levels in the same healthy subjects, with mediation results supporting the model that loss of prefrontal inhibition of the amygdala leads to its over-activity and generation of negative affect. Given that the present findings in healthy subjects are consistent with evidence for similar or related changes in this circuitry in mood and anxiety disorders [3], [5], [9], [24], [66], these results provide further support for the continuum/dimensional model of these disorders [50]–[52], [67].

## References

[1] Jackson J (1931–1932) In: Taylor J, editor. Selected writings of John Hughlings Jackson. London: Hodder.

[2] Ey H (1978) Hughlings Jackson’s fundamental principles applied to psychiatry. In: R H, editor. Historical Explorations in Medicine and Psychiatry. New York: Springer. 204–219.

[3] Mayberg HS (2003) Modulating dysfunctional limbic-cortical circuits in depression: towards development of brain-based algorithms for diagnosis and optimised treatment. Br Med Bull 65: 193–207. 12697626 10.1093/bmb/65.1.193

[4] Davidson RJ (2002) Anxiety and affective style: role of prefrontal cortex and amygdala. Biol Psychiatry 51: 68–80. 11801232 10.1016/s0006-3223(01)01328-2

[5] Shin LM, Rauch SL, Pitman RK (2006) Amygdala, medial prefrontal cortex, and hippocampal function in PTSD. Ann N Y Acad Sci 1071: 67–79. 16891563 10.1196/annals.1364.007

[6] Williams LM, Das P, Harris AW, Liddell BB, Brammer MJ, et al (2004) Dysregulation of arousal and amygdala-prefrontal systems in paranoid schizophrenia. Am J Psychiatry 161: 480–489. 14992974 10.1176/appi.ajp.161.3.480

[7] Laruelle M, Kegeles LS, Abi-Dargham A (2003) Glutamate, dopamine, and schizophrenia: from pathophysiology to treatment. Ann N Y Acad Sci 1003: 138–158. 14684442 10.1196/annals.1300.063

[8] Mayberg HS, Liotti M, Brannan SK, McGinnis S, Mahurin RK, et al (1999) Reciprocal limbic-cortical function and negative mood: converging PET findings in depression and normal sadness. Am J Psychiatry 156: 675–682. 10327898 10.1176/ajp.156.5.675

[9] Almeida JR, Versace A, Mechelli A, Hassel S, Quevedo K, et al (2009) Abnormal amygdala-prefrontal effective connectivity to happy faces differentiates bipolar from major depression. Biol Psychiatry 66: 451–459. 19450794 10.1016/j.biopsych.2009.03.024 PMC2740996

[10] Foland LC, Altshuler LL, Bookheimer SY, Eisenberger N, Townsend J, et al (2008) Evidence for deficient modulation of amygdala response by prefrontal cortex in bipolar mania. Psychiatry Res 162: 27–37. 18063349 10.1016/j.pscychresns.2007.04.007 PMC2410029

[11] Shin LM, Wright CI, Cannistraro PA, Wedig MM, McMullin K, et al (2005) A functional magnetic resonance imaging study of amygdala and medial prefrontal cortex responses to overtly presented fearful faces in posttraumatic stress disorder. Arch Gen Psychiatry 62: 273–281. 15753240 10.1001/archpsyc.62.3.273

[12] Watson D, Clark LA (1984) Negative affectivity: the disposition to experience aversive emotional states. Psychol Bull 96: 465–490. 6393179

[13] Bishop SJ (2007) Neurocognitive mechanisms of anxiety: an integrative account. Trends Cogn Sci 11: 307–316. 17553730 10.1016/j.tics.2007.05.008

[14] Drevets WC, Videen TO, Price JL, Preskorn SH, Carmichael ST, et al (1992) A functional anatomical study of unipolar depression. J Neurosci 12: 3628–3641. 1527602 10.1523/JNEUROSCI.12-09-03628.1992 PMC6575746

[15] Abercrombie HC, Schaefer SM, Larson CL, Oakes TR, Lindgren KA, et al (1998) Metabolic rate in the right amygdala predicts negative affect in depressed patients. Neuroreport 9: 3301–3307. 9831467 10.1097/00001756-199810050-00028

[16] Pezawas L, Meyer-Lindenberg A, Drabant EM, Verchinski BA, Munoz KE, et al (2005) 5-HTTLPR polymorphism impacts human cingulate-amygdala interactions: a genetic susceptibility mechanism for depression. Nat Neurosci 8: 828–834. 15880108 10.1038/nn1463

[17] Kim MJ, Gee DG, Loucks RA, Davis FC, Whalen PJ (2011) Anxiety dissociates dorsal and ventral medial prefrontal cortex functional connectivity with the amygdala at rest. Cereb Cortex 21: 1667–1673. 21127016 10.1093/cercor/bhq237 PMC3116741

[18] Kim MJ, Whalen PJ (2009) The structural integrity of an amygdala-prefrontal pathway predicts trait anxiety. J Neurosci 29: 11614–11618. 19759308 10.1523/JNEUROSCI.2335-09.2009 PMC2791525

[19] Biswal B, Yetkin FZ, Haughton VM, Hyde JS (1995) Functional connectivity in the motor cortex of resting human brain using echo-planar MRI. Magn Reson Med 34: 537–541. 8524021 10.1002/mrm.1910340409

[20] Detre JA, Zhang W, Roberts DA, Silva AC, Williams DS, et al (1994) Tissue specific perfusion imaging using arterial spin labeling. NMR Biomed 7: 75–82. 8068529 10.1002/nbm.1940070112

[21] Wang Y, Saykin AJ, Pfeuffer J, Lin C, Mosier KM, et al (2011) Regional reproducibility of pulsed arterial spin labeling perfusion imaging at 3T. Neuroimage 54: 1188–1195. 20800097 10.1016/j.neuroimage.2010.08.043 PMC2997151

[22] Fox MD, Raichle ME (2007) Spontaneous fluctuations in brain activity observed with functional magnetic resonance imaging. Nat Rev Neurosci 8: 700–711. 17704812 10.1038/nrn2201

[23] Roy AK, Shehzad Z, Margulies DS, Kelly AM, Uddin LQ, et al (2009) Functional connectivity of the human amygdala using resting state fMRI. Neuroimage 45: 614–626. 19110061 10.1016/j.neuroimage.2008.11.030 PMC2735022

[24] Etkin A, Prater KE, Schatzberg AF, Menon V, Greicius MD (2009) Disrupted amygdalar subregion functional connectivity and evidence of a compensatory network in generalized anxiety disorder. Arch Gen Psychiatry 66: 1361–1372. 19996041 10.1001/archgenpsychiatry.2009.104 PMC12553334

[25] Amaral DG, Price JL (1984) Amygdalo-cortical projections in the monkey (Macaca fascicularis). J Comp Neurol 230: 465–496. 6520247 10.1002/cne.902300402

[26] Barbas H, De Olmos J (1990) Projections from the amygdala to basoventral and mediodorsal prefrontal regions in the rhesus monkey. J Comp Neurol 300: 549–571. 2273093 10.1002/cne.903000409

[27] First MB, Spitzer RL, Gibbon M, Williams JBW (1995) Structured Clinical Interview for the DSM-IV: Axis I Disorders. New York: New York State Psychiatric Institue, Biometrics Research.

[28] Spielberger C, Gorsuch R, Lushene R, Vagg P, Jacobs G (1983) Manual for the State-Trait Anxiety Inventory. Palo Alto, CA: Consulting Psychologists Press.

[29] Caci H, Bayle FJ, Dossios C, Robert P, Boyer P (2003) The Spielberger Trait Anxiety Inventory measures more than anxiety. Eur Psychiatry 18: 394–400. 14680715 10.1016/j.eurpsy.2003.05.003

[30] Kohn PM, Kantor L, DeCicco TL, Beck AT (2008) The Beck Anxiety Inventory-Trait (BAIT): a measure of dispositional anxiety not contaminated by dispositional depression. J Pers Assess 90: 499–506. 18704809 10.1080/00223890802248844

[31] Gros DF, Antony MM, Simms LJ, McCabe RE (2007) Psychometric properties of the State-Trait Inventory for Cognitive and Somatic Anxiety (STICSA): comparison to the State-Trait Anxiety Inventory (STAI). Psychol Assess 19: 369–381. 18085930 10.1037/1040-3590.19.4.369

[32] Bados A, Gomez-Benito J, Balaguer G (2010) The state-trait anxiety inventory, trait version: does it really measure anxiety? J Pers Assess 92: 560–567. 20954057 10.1080/00223891.2010.513295

[33] Bieling PJ, Antony MM, Swinson RP (1998) The State-Trait Anxiety Inventory, Trait version: structure and content re-examined. Behav Res Ther 36: 777–788. 9682533 10.1016/s0005-7967(98)00023-0

[34] Kvaal K, Ulstein I, Nordhus IH, Engedal K (2005) The Spielberger State-Trait Anxiety Inventory (STAI): the state scale in detecting mental disorders in geriatric patients. International journal of geriatric psychiatry 20: 629–634. 16021666 10.1002/gps.1330

[35] Luh WM, Wong EC, Bandettini PA, Hyde JS (1999) QUIPSS II with thin-slice TI1 periodic saturation: a method for improving accuracy of quantitative perfusion imaging using pulsed arterial spin labeling. Magn Reson Med 41: 1246–1254. 10371458 10.1002/(sici)1522-2594(199906)41:6<1246::aid-mrm22>3.0.co;2-n

[36] Wasan AD, Loggia ML, Chen LQ, Napadow V, Kong J, et al (2011) Neural correlates of chronic low back pain measured by arterial spin labeling. Anesthesiology 115: 364–374. 21720241 10.1097/ALN.0b013e318220e880 PMC3286828

[37] Amunts K, Kedo O, Kindler M, Pieperhoff P, Mohlberg H, et al (2005) Cytoarchitectonic mapping of the human amygdala, hippocampal region and entorhinal cortex: intersubject variability and probability maps. Anat Embryol (Berl) 210: 343–352. 16208455 10.1007/s00429-005-0025-5

[38] Woolrich MW, Jbabdi S, Patenaude B, Chappell M, Makni S, et al (2009) Bayesian analysis of neuroimaging data in FSL. Neuroimage 45: S173–186. 19059349 10.1016/j.neuroimage.2008.10.055

[39] Jenkinson M, Bannister P, Brady M, Smith S (2002) Improved optimization for the robust and accurate linear registration and motion correction of brain images. Neuroimage 17: 825–841. 12377157 10.1016/s1053-8119(02)91132-8

[40] Greve DN, Fischl B (2009) Accurate and robust brain image alignment using boundary-based registration. Neuroimage 48: 63–72. 19573611 10.1016/j.neuroimage.2009.06.060 PMC2733527

[41] Friston KJ, Worsley KJ, Frackowiak RSJ, Mazziotta JC, Evans AC (1993) Assessing the significance of focal activations using their spatial extent. Hum Brain Mapp: 210–220. 10.1002/hbm.460010306 24578041

[42] Van Dijk KR, Hedden T, Venkataraman A, Evans KC, Lazar SW, et al (2010) Intrinsic functional connectivity as a tool for human connectomics: theory, properties, and optimization. J Neurophysiol 103: 297–321. 19889849 10.1152/jn.00783.2009 PMC2807224

[43] Van Dijk KR, Sabuncu MR, Buckner RL (2012) The influence of head motion on intrinsic functional connectivity MRI. Neuroimage 59: 431–438. 21810475 10.1016/j.neuroimage.2011.07.044 PMC3683830

[44] Zar JH (1996) Biostatistical analysis 3rd edn. Practice Hall, New Jersey.

[45] Roy M, Shohamy D, Wager TD (2012) Ventromedial prefrontal-subcortical systems and the generation of affective meaning. Trends Cogn Sci 16: 147–156. 22310704 10.1016/j.tics.2012.01.005 PMC3318966

[46] Tailarach J, Tournoux P (1988) Co-planar Stereotaxic Atlas of the Human Brain. Rayport M, translator. New York, NY: Thieme Medical Publishers.

[47] Maldjian JA, Laurienti PJ, Kraft RA, Burdette JH (2003) An automated method for neuroanatomic and cytoarchitectonic atlas-based interrogation of fMRI data sets. Neuroimage 19: 1233–1239. 12880848 10.1016/s1053-8119(03)00169-1

[48] Preacher KJ, Hayes AF (2008) Asymptotic and resampling strategies for assessing and comparing indirect effects in multiple mediator models. Behav Res Methods 40: 879–891. 18697684 10.3758/brm.40.3.879

[49] Kennedy BL, Schwab JJ, Morris RL, Beldia G (2001) Assessment of state and trait anxiety in subjects with anxiety and depressive disorders. Psychiatric Quarterly 72: 263–276. 11467160 10.1023/a:1010305200087

[50] Flett GL, Vredenburg K, Krames L (1997) The continuity of depression in clinical and nonclinical samples. Psychol Bull 121: 395–416. 9136642 10.1037/0033-2909.121.3.395

[51] McGorry PD, Nelson B, Goldstone S, Yung AR (2010) Clinical staging: a heuristic and practical strategy for new research and better health and social outcomes for psychotic and related mood disorders. Can J Psychiatry 55: 486–497. 20723276 10.1177/070674371005500803

[52] Preisig M, Merikangas KR, Angst J (2001) Clinical significance and comorbidity of subthreshold depression and anxiety in the community. Acta Psychiatr Scand 104: 96–103. 11473502 10.1034/j.1600-0447.2001.00284.x

[53] Fischer H, Tillfors M, Furmark T, Fredrikson M (2001) Dispositional pessimism and amygdala activity: a PET study in healthy volunteers. Neuroreport 12: 1635–1638. 11409730 10.1097/00001756-200106130-00024

[54] Somerville LH, Kim H, Johnstone T, Alexander AL, Whalen PJ (2004) Human amygdala responses during presentation of happy and neutral faces: correlations with state anxiety. Biol Psychiatry 55: 897–903. 15110733 10.1016/j.biopsych.2004.01.007

[55] Bishop SJ, Duncan J, Lawrence AD (2004) State anxiety modulation of the amygdala response to unattended threat-related stimuli. J Neurosci 24: 10364–10368. 15548650 10.1523/JNEUROSCI.2550-04.2004 PMC6730310

[56] Etkin A, Klemenhagen KC, Dudman JT, Rogan MT, Hen R, et al (2004) Individual differences in trait anxiety predict the response of the basolateral amygdala to unconsciously processed fearful faces. Neuron 44: 1043–1055. 15603746 10.1016/j.neuron.2004.12.006

[57] Ebeling U, von Cramon D (1992) Topography of the uncinate fascicle and adjacent temporal fiber tracts. Acta Neurochir (Wien) 115: 143–148. 1605083 10.1007/BF01406373

[58] Lehman JF, Greenberg BD, McIntyre CC, Rasmussen SA, Haber SN (2011) Rules ventral prefrontal cortical axons use to reach their targets: implications for diffusion tensor imaging tractography and deep brain stimulation for psychiatric illness. J Neurosci 31: 10392–10402. 21753016 10.1523/JNEUROSCI.0595-11.2011 PMC3445013

[59] Holmes AJ, Lee PH, Hollinshead MO, Bakst L, Roffman JL, et al (2012) Individual Differences in Amygdala-Medial Prefrontal Anatomy Link Negative Affect, Impaired Social Functioning, and Polygenic Depression Risk. J Neurosci 32: 18087–18100. 23238724 10.1523/JNEUROSCI.2531-12.2012 PMC3674506

[60] Sotres-Bayon F, Quirk GJ (2010) Prefrontal control of fear: more than just extinction. Curr Opin Neurobiol 20: 231–235. 20303254 10.1016/j.conb.2010.02.005 PMC2878722

[61] Quirk GJ, Likhtik E, Pelletier JG, Pare D (2003) Stimulation of medial prefrontal cortex decreases the responsiveness of central amygdala output neurons. J Neurosci 23: 8800–8807. 14507980 10.1523/JNEUROSCI.23-25-08800.2003 PMC6740415

[62] Phelps EA, Delgado MR, Nearing KI, LeDoux JE (2004) Extinction learning in humans: role of the amygdala and vmPFC. Neuron 43: 897–905. 15363399 10.1016/j.neuron.2004.08.042

[63] Kim H, Somerville LH, Johnstone T, Alexander AL, Whalen PJ (2003) Inverse amygdala and medial prefrontal cortex responses to surprised faces. Neuroreport 14: 2317–2322. 14663183 10.1097/00001756-200312190-00006

[64] Milad MR, Wright CI, Orr SP, Pitman RK, Quirk GJ, et al (2007) Recall of fear extinction in humans activates the ventromedial prefrontal cortex and hippocampus in concert. Biol Psychiatry 62: 446–454. 17217927 10.1016/j.biopsych.2006.10.011

[65] Royer S, Pare D (2002) Bidirectional synaptic plasticity in intercalated amygdala neurons and the extinction of conditioned fear responses. Neuroscience 115: 455–462. 12421611 10.1016/s0306-4522(02)00455-4

[66] Drevets WC (1999) Prefrontal cortical-amygdalar metabolism in major depression. Ann N Y Acad Sci 877: 614–637. 10415674 10.1111/j.1749-6632.1999.tb09292.x

[67] Hankin BL, Fraley RC, Lahey BB, Waldman ID (2005) Is depression best viewed as a continuum or discrete category? A taxometric analysis of childhood and adolescent depression in a population-based sample. J Abnorm Psychol 114: 96–110. 15709816 10.1037/0021-843X.114.1.96

[68] Öngür D, Ferry AT, Price JL (2003) Architectonic subdivision of the human orbital and medial prefrontal cortex. Journal of Comparative Neurology 460: 425–449. 12692859 10.1002/cne.10609

[69] Mayberg HS, Brannan SK, Tekell JL, Silva JA, Mahurin RK, et al (2000) Regional metabolic effects of fluoxetine in major depression: serial changes and relationship to clinical response. Biol Psychiatry 48: 830–843. 11063978 10.1016/s0006-3223(00)01036-2

[70] Mayberg HS, Lozano AM, Voon V, McNeely HE, Seminowicz D, et al (2005) Deep brain stimulation for treatment-resistant depression. Neuron 45: 651–660. 15748841 10.1016/j.neuron.2005.02.014

[71] Pizzagalli D, Oakes T, Fox A, Chung M, Larson C, et al (2004) Functional but not structural subgenual prefrontal cortex abnormalities in melancholia. Mol Psychiatry 9: 393–405. 10.1038/sj.mp.4001501 14699431

[72] Kumano H, Ida I, Oshima A, Takahashi K, Yuuki N, et al (2007) Brain metabolic changes associated with predispotion to onset of major depressive disorder and adjustment disorder in cancer patients–A preliminary PET study. J Psychiatr Res 41: 591–599. 16684544 10.1016/j.jpsychires.2006.03.006

[73] Nahas Z, Teneback C, Chae J-H, Mu Q, Molnar C, et al (2007) Serial vagus nerve stimulation functional MRI in treatment-resistant depression. Neuropsychopharmacology 32: 1649–1660. 17203016 10.1038/sj.npp.1301288

[74] Blair JR, Spreen O (1989) Predicting premorbid IQ: a revision of the National Adult Reading Test. The Clinical Neuropsychologist 3: 129–136.

[75] Hollingshead A (1957) Two factor index of social position.

[76] Beck AT, Ward CH, Mendelson M, Mock J, Erbaugh J (1961) An inventory for measuring depression. Arch Gen Psychiatry 4: 561. 13688369 10.1001/archpsyc.1961.01710120031004

